# Prescribing in First Episode Psychosis

**DOI:** 10.1192/bjo.2024.429

**Published:** 2024-08-01

**Authors:** Edita Searle, Raeema Patel, Robertson Macpherson

**Affiliations:** Gloucestershire Health and Care Trust, Gloucester, United Kingdom

## Abstract

**Aims:**

This audit aimed to review prescribing in First Episode Psychosis (FEP) in Gloucestershire Health and Care NHS Trust, against NICE guidelines (CG 178) we hoped to develop prescribing guidelines for the Trust and to compare our results with Avon and Wiltshire Partnership (AWP) Trust's results of similar audit (AWP-235 Audit of Prescribing in FEP).

**Methods:**

The sample was the Trust Early intervention (EI) caseload of patients with diagnosis FEP. We developed the audit tool based on AWP's audit methodology.

We gathered information about

- The role of initial prescribers.
- The prescribing of up to three antipsychotics.
- Choices of antipsychotic medication, whether the patient was given choice and information about the antipsychotic.
- Recorded reviews of side effects.
- Duration of treatment.
- Reasons for switching antipsychotic.
- Whether clozapine was offered to patients where indicated.
- Whether a recommended antipsychotic free period allowing for investigations and assessments was adhered to.
- Other medications prescribed alongside the antipsychotics.

**Results:**

77 patients were identified.

- Adherence to the NICE guideline criterion of initial prescriber being in secondary care was good.
- Olanzapine was the preferred first antipsychotic choice for 50% of patients, aripiprazole was the most common choice as 2nd and 3rd antipsychotic (around 30% patients).
- Recording of Information about antipsychotic treatment was lower than expected, about 30% of the sample at first choice, this increased to 50% for second choice and 40% at the third choice of antipsychotic.
- Around 90% of the sample had recorded review of medication and its side effects.
- 17% of the sample had duration of treatment less than 6 weeks at first antipsychotic, this dropped to 9% and 6% at second and third respectively.
- Reasons for switching were mostly due to side effects and lack of efficacy. Refusal to take the antipsychotic was a common reason for switching to the third antipsychotic.
- Only about 20% of patients who were eligible were offered clozapine.
- An antipsychotic free period up to 7 days was adhered to in almost 70%.

**Conclusion:**

As a result of the audit findings we have developed Trust prescribing guidelines for adults presenting with FEP, which include recommendation for 7-day antipsychotic free assessment period, need to involve patients and family/carers when making decisions about choice of medication and recorded discussion about clozapine for eligible patients.

